# Vernier-Templated Synthesis, Crystal Structure, and Supramolecular Chemistry of a 12-Porphyrin Nanoring

**DOI:** 10.1002/chem.201403714

**Published:** 2014-08-25

**Authors:** Dmitry V Kondratuk, Johannes K Sprafke, Melanie C O'Sullivan, Luis M A Perdigao, Alex Saywell, Marc Malfois, James N O'Shea, Peter H Beton, Amber L Thompson, Harry L Anderson

**Affiliations:** [a] Department of Chemistry, University of Oxford, Chemistry Research Laboratory Oxford, OX1 3TA (UK) E-mail: amber.thompson@chem.ox.ac.uk harry.anderson@chem.ox.ac.uk; [b] School of Physics & Astronomy, University of Nottingham Nottingham, NG7 2RD (UK); [c] Diamond Light Source Ltd. Harwell Science and Innovation Campus Didcot, OX11 0DE (UK)

**Keywords:** conjugation, macrocycles, porphyrinoids, supramolecular chemistry, template synthesis

## Abstract

Vernier templating exploits a mismatch between the number of binding sites in a template and a reactant to direct the formation of a product that is large enough to bind several template units. Here, we present a detailed study of the Vernier-templated synthesis of a 12-porphyrin nanoring. NMR and small-angle X-ray scattering (SAXS) analyses show that Vernier complexes are formed as intermediates in the cyclo-oligomerization reaction. UV/Vis/NIR titrations show that the three-component assembly of the 12-porphyrin nanoring figure-of-eight template complex displays high allosteric cooperativity and chelate cooperativity. This nanoring–template 1:2 complex is among the largest synthetic molecules to have been characterized by single-crystal analysis. It crystallizes as a racemate, with an angle of 27° between the planes of the two template units. The crystal structure reveals many unexpected intramolecular C–H⋅⋅⋅N contacts involving the *tert*-butyl side chains. Scanning tunneling microscopy (STM) experiments show that molecules of the 12-porphyrin template complex can remain intact on the gold surface, although the majority of the material unfolds into the free nanoring during electrospray deposition.

## Introduction

Ever since Sondheimer’s seminal work on annulenes,[1] macrocycles with π-conjugated perimeters have provided fascinating systems for testing theories of molecular electronic structure. Recently, the invention of synthetic routes to very large π-conjugated macrocycles has sparked a renaissance in this field, driven by the quest to understand energy transfer, charge delocalization, and nonlinear optical phenomena in these nanostructures.[2–15] Template-directed synthesis makes it possible to create large, fully π-conjugated macrocycles in a size-range that could not have been reached without programmed self-assembly.[7, 9, 10] The classical template effect translates information from the size and shape of a template to direct the construction of a complementary macrocycle.[16] We have used this approach to prepare nanorings consisting of 6 and 8 porphyrin units, using hexadentate and octadentate templates.[9a–c] This classical approach is not convenient for the synthesis of larger nanorings because of the inaccessibility of suitable templates.

**Scheme 1:**
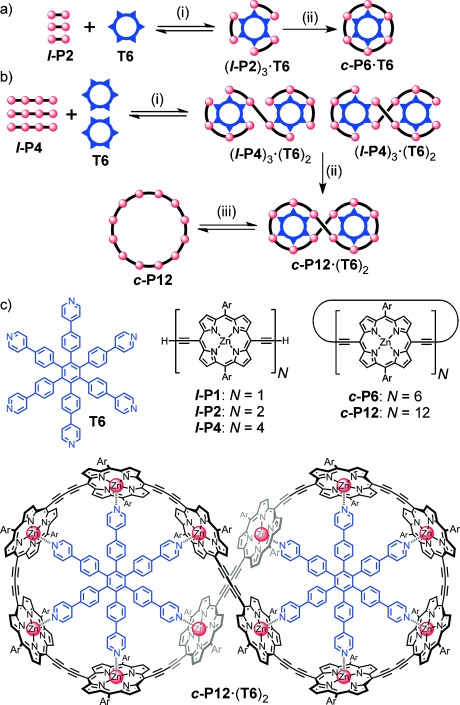
a) Classical template-directed synthesis of *c*-P6. b) Vernier-templated synthesis of *c*-P12: (i) self-assembly; (ii) [PdCl_2_(PPh_3_)_2_], CuI, benzoquinone, *i*Pr_2_NH; (iii) pyridine. c) Chemical structures; Ar=3,5-bis(*tert*-butyl)phenyl or 3,5-bis(octyloxy)phenyl, as indicated by the subscript “*t*Bu” or “C8”, respectively.

Vernier complexes are formed between a host and a guest when the number of binding sites on one component is not an integer multiple of the number of binding sites on the other component. Self-assembly generates a structure with a number of binding sites that is the lowest common multiple of the numbers of sites on the host and the guest.[17] Recently, we demonstrated that the Vernier effect can be exploited to direct the synthesis of large nanorings using small templates.[9d,e] In effect, the size of the template can be amplified if the number of binding sites on the template is not a multiple of the number of binding sites on the building block. This concept was first illustrated by the synthesis of a 12-porphyrin nanoring ***c*****-P12** by coupling a linear porphyrin tetramer ***l*****-P4** in the presence of a hexadentate template **T6** (Scheme 1).[9d] Here, we present a full account of the synthesis, crystal structure, and template-binding behavior of ***c*****-P12**, including an investigation into the mechanism of Vernier templating. Small-angle X-ray scattering (SAXS) and NMR spectroscopic analysis provide evidence for the formation of the Vernier complex (***l*****-P4**)_3_**⋅**(**T6**)_2_ under the conditions of the template-directed synthesis. UV/Vis/NIR titrations show that folding of ***c*****-P12** into the figure-of-eight template complex ***c*****-P12⋅**(**T6**)_2_ is a highly cooperative process. Here, we report the crystal structure of ***c*****-P12⋅**(**T6**)_2_, which is the largest porphyrin oligomer yet to have been characterized by single-crystal X-ray analysis. Scanning tunneling microscopy (STM) was also used to image ***c*****-P12** and ***c*****-P12⋅**(**T6**)_2_ molecules on a gold surface.

## Results and Discussion

### Synthesis of *c*-P12

**Figure 1:**
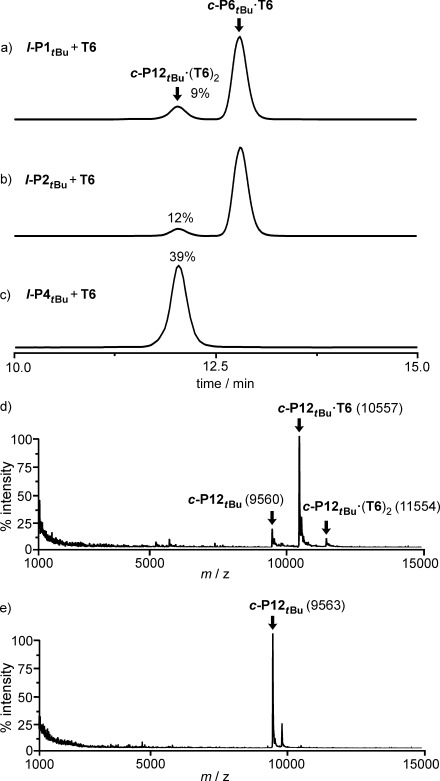
Analytical GPC traces (THF, detection at 360 nm) of the crude reaction mixtures of coupling a) *l*-P1_*t*Bu_, b) *l*-P2_*t*Bu_, and c) *l*-P4_*t*Bu_ in the presence of T6 and the corresponding analytical yields. The analytical yields shown were determined by comparing the areas of *c*-P12_*t*Bu_⋅(T6)_2_ with the area of standard injection of *c*-P12_*t*Bu_⋅(T6)_2_. Before GPC analysis, the coupling reagents (the catalysts and 1,4-benzoquinone) and insoluble polymers were removed by passing through a short alumina column in CHCl_3_. MALDI-TOF spectra of d) *c*-P12_*t*Bu_⋅(T6)_2_ and e) *c*-P12_*t*Bu_.

During our initial work on the synthesis of the cyclic porphyrin hexamer ***c*****-P6**_***t*****Bu**_, by palladium-catalyzed oxidative coupling of the linear porphyrin monomer ***l*****-P1**_***t*****Bu**_ or dimer ***l*****-P2**_***t*****Bu**_ in the presence of the hexapyridyl template **T6** (Scheme 1a), we noticed the formation of a high-mass byproduct, which was identified as the 12-porphyrin nanoring figure-of-eight complex ***c*****-P12**_***t*****Bu**_**⋅**(**T6**)_2_.[9b,c] Analytical gel permeation chromatography (GPC) analysis of crude reaction mixtures (Figure 1 a,b) indicated that ***c*****-P12**_***t*****Bu**_**⋅**(**T6**)_2_ was formed in yields of 9 and 12 % from ***l*****-P1**_***t*****Bu**_ and ***l*****-P2**_***t*****Bu**_, respectively. The mass spectrum of ***c*****-P12**_***t*****Bu**_**⋅**(**T6**)_2_ (MALDI-TOF MS; Figure 1 d) reveals a molecular ion at twice the molecular weight of ***c*****-P6**_***t*****Bu**_**⋅T6**, as well as peaks related to loss of one or two template units. Treatment with pyridine, gave the free nanoring ***c*****-P12**_***t*****Bu**_, which was thoroughly characterized by ^1^H NMR spectroscopy and MALDI-TOF MS analysis (Figure 1 e).[9d]

These serendipitous syntheses of the 12-ring ***c*****-P12**_***t*****Bu**_ from porphyrin monomer and dimer indicated that a porphyrin tetramer starting material ***l*****-P4**_***t*****Bu**_ would give the 12-ring as the main product, because the 6-ring ***c*****-P6**_***t*****Bu**_ could not be formed in this case. We conjectured that a Vernier complex (***l*****-P4**_***t*****Bu**_)_3_**⋅**(**T6**)_2_ might form directly from the starting materials and lead to efficient formation of the figure-of-eight complex ***c*****-P12**_***t*****Bu**_**⋅**(**T6**)_2_ (Scheme 1b). Alternatively, oligomerization of the unbound porphyrin tetramer and subsequent cyclization around two, four, six etc. template molecules should form the series of macrocycles ***c*****-P*N*** with ***N*** being a multiple of twelve. In general, the coupling of a starting material with *x* binding sites in the presence of a suitable template with *y* binding sites should lead to formation of a macrocycle with *z* binding sites, where *z* is lowest common multiple of *x* and *y*.

As expected, palladium-catalyzed oxidative coupling of the linear porphyrin tetramer ***l*****-P4**_***t*****Bu**_ in the presence of **T6** gave the figure-of-eight complex ***c*****-P12**_***t*****Bu**_**⋅**(**T6**)_2_ as the major product in 39 % isolated yield (Figure 1 c).[9d] The only other products of this reaction were insoluble polymers and traces of high-mass oligomers, which were difficult to isolate due to their low solubility. To learn more about this reaction, we investigated the coupling of porphyrin tetramer bearing octyloxy side chains ***l*****-P4_C8_**, as a means to improve the solubility of cyclic byproducts.

**Figure 2:**
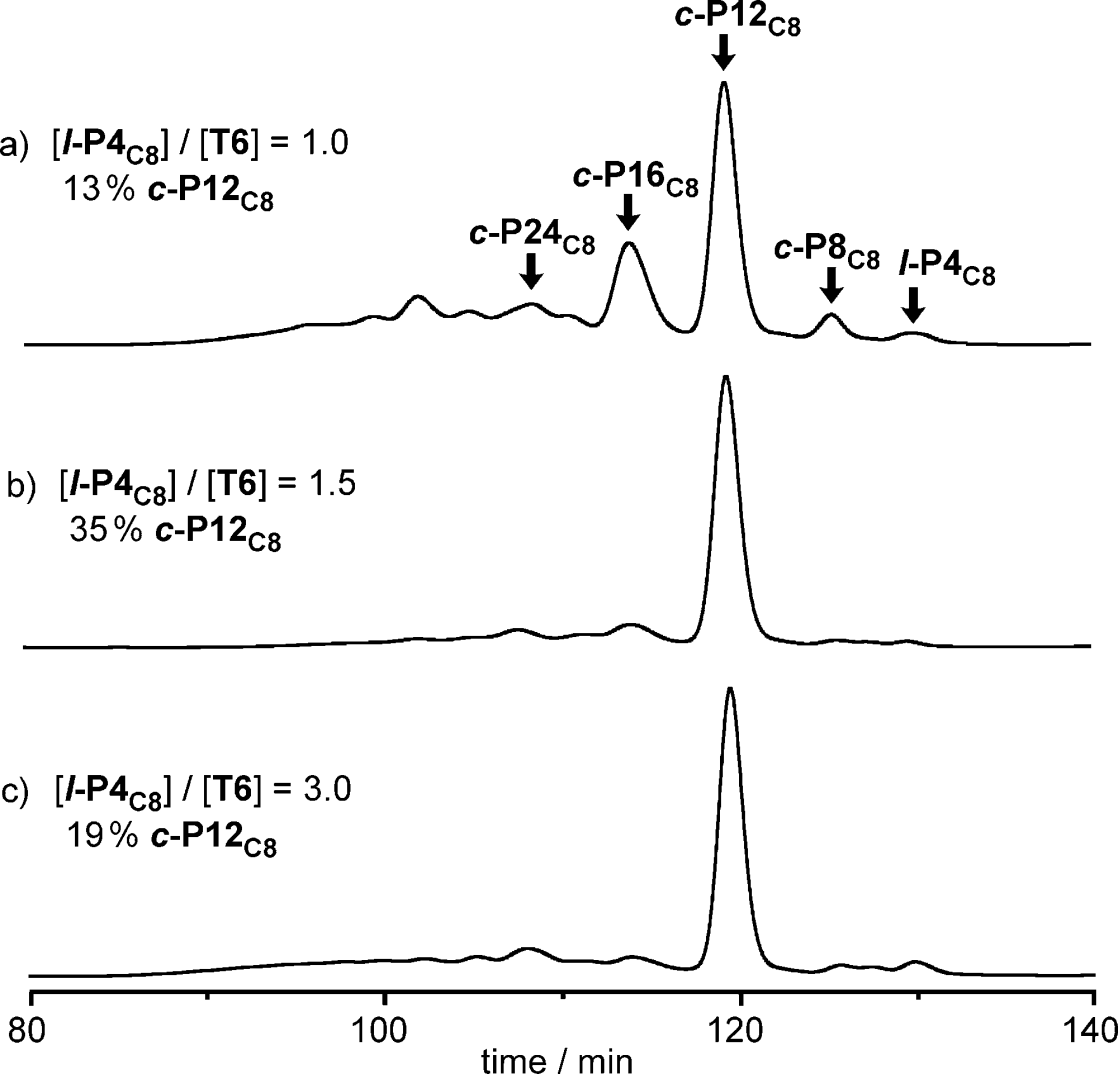
Recycling GPC traces (2nd cycle shown, toluene/1 % pyridine, detection at 500 nm) of the crude reaction mixtures of coupling *l*-P4_C8_ in the presence of T6 at various *l*-P4_C8_/T6 ratios and the corresponding analytical yields of *c*-P12_C8_. The analytical yields were determined by comparing the areas of *c*-P12_C8_ with the area of standard injection of *c*-P12_C8_. The coupling reagents (the catalysts and 1,4-benzoquinone) and T6 were removed by passing through a short alumina column in CHCl_3_ and a size-exclusion column in CHCl_3_/10 % pyridine, respectively.

Coupling of the linear porphyrin tetramer ***l*****-P4_C8_** in the presence of template **T6** at various mole ratios (***l*****-P4_C8_**:**T6**) gave mixtures of cyclic and linear oligomers, all as complexes with the **T6** template. The linear polymers were removed by using a short alumina column, and the template was removed by addition of pyridine, prior to GPC analysis (Figure 2). In all cases studied (***l*****-P4_C8_**/**T6**=1.0, 1.5, 3.0), the major product was ***c*****-P12_C8_**. Formation of the 12-ring is most efficient when using a stoichiometric amount of template (***l*****-P4_C8_**/**T6**=1.5). However, ***c*****-P12_C8_** was never the only product and traces of smaller (***c*****-P8_C8_**) and larger (e.g., ***c*****-P16_C8_** and ***c*****-24_C8_**) cyclic species were detected. None of these cyclic oligomers formed in the absence of a template.

In keeping with the GPC analysis, ***c*****-P12_C8_** was isolated in 32 % yield by using a stoichiometric amount of **T6**. This yield is comparable to the isolated yield obtained from the Vernier synthesis of ***c*****-P12**_***t*****Bu**_ (39 %). With a 1:1 mole ratio of ***l*****-P4_C8_**:**T6**, the yield of ***c*****-P12_C8_** decreased to 16 %, and ***c*****-P16_C8_** was isolated in 6 % yield.

### Probing the Mechanism of Vernier Templating

In principle, Vernier templated coupling of a starting material ***l*****-P*x*** with *x* binding sites in the presence of a template **T*y*** to give a product ***c-*****P*z*** (where *z* is the lowest common multiple of *x* and *y*) could operate through two mechanisms: 1) the template waits until oligomerization has generated a linear species ***l*****-P*z***, at which point it binds strongly to form a complex ***l*****-P*z*⋅**(**T*y***)_*z*/*y*_, which then undergoes rapid coupling to give ***c*****-P*z*⋅**(**T*y***)_*z*/*y*_, or 2) a Vernier complex (***l*****-P*x***)_*z*/*x*_**⋅**(**T*y***)_*z*/*y*_ is formed, which then couples to give ***c*****-P*z*⋅**(**T*y***)_*z*/*y*_. In practice, the reaction could proceed by a combination of these extremes, with coupling of both free and bound oligomers. We decided to test whether ***l*****-P4**_***t*****Bu**_ coordinates to **T6** to form a stable Vernier complex (***l*****-P4**)_3_**⋅**(**T6**)_2_ under the conditions of the reaction (toluene solution, 20 °C), to establish whether this complex is a plausible intermediate.

A ^1^H NMR titration of ***l*****-P4_C8_** with **T6** (500 MHz, CDCl_3_, 298 K) showed only broadening of the initial spectrum of ***l*****-P4_C8_** and no useful structural information could be extracted. To assess the size of the complex, we used diffusion-ordered NMR spectroscopy (DOSY).[18] The 2D DOSY spectrum of a 3:2 mixture of ***l*****-P4_C8_** and **T6** shows similar diffusion coefficients for porphyrin and template signals, thereby confirming that both components bind together to form a complex (Figure S3). The diffusion coefficient of this complex (*D*=1.92±0.14×10^−10^ m^2^ s^−1^) is the same as that of the figure-of-eight ***c*****-P12_C8_⋅**(**T6**)_2_ complex (*D*=1.91±0.07×10^−10^ m^2^ s^−1^), strongly supporting the formation of a Vernier complex (***l*****-P4_C8_**)_3_**⋅**(**T6**)_2_. The diffusion coefficients of ***c*****-P12_C8_⋅**(**T6**)_2_ and (***l*****-P4_C8_**)_3_**⋅**(**T6**)_2_ are significantly smaller than those of ***l*****-P4_C8_** (*D*=2.52±0.04×10^−10^ m^2^ s^−1^) and **T6** (*D*=5.34±0.25×10^−10^ m^2^ s^−1^) and slightly bigger than that of ***c*****-P12_C8_** (*D*=1.58±0.04×10^−10^ m^2^ s^−1^), all measured at 298 K in CDCl_3_ (with 1 % *d*_5_-pyridine to prevent aggregation in the case of ***l*****-P4_C8_** and ***c*****-P12_C8_**).

**Figure 3:**
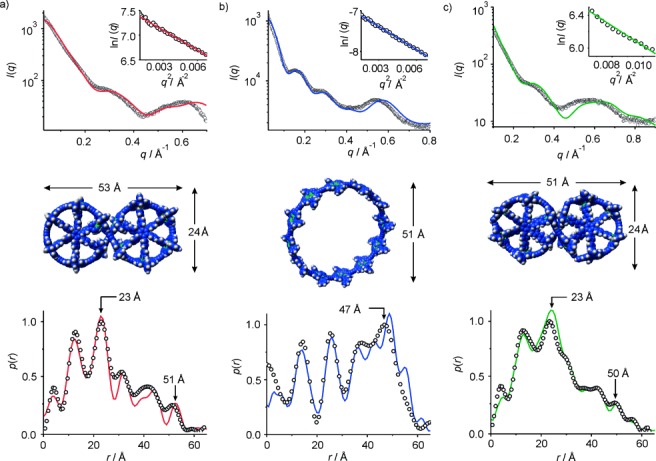
SAXS analysis of a) figure-of-eight complex *c*-P12_*t*Bu_⋅(T6)_2_ in toluene, b) cyclic dodecamer *c*-P12_*t*Bu_ in toluene/1 % pyridine, and c) Vernier complex (*l*-P4_*t*Bu_)_3_⋅(T6)_2_ in toluene (298 K). The top row shows the experimental scattering data (black circles) together with the simulated curves based on calculated models (solid lines) calculated from the experimental scattering data and the radii of gyration *R*_g_. The bottom row shows pair-distribution functions determined experimentally (black circles) and from models (solid lines).

**Figure 4:**
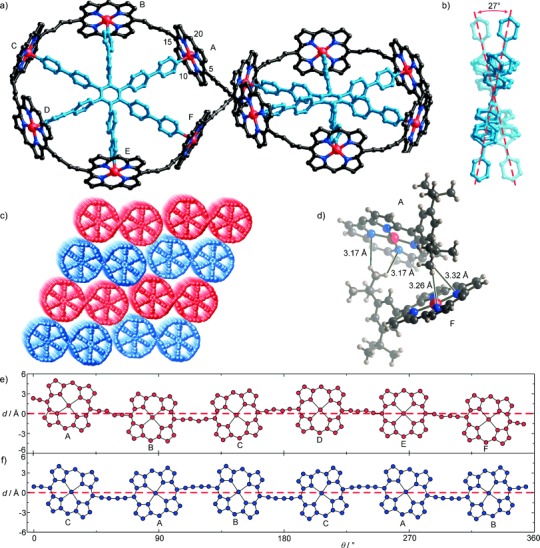
a) Solid-state structure of *c*-P12_*t*Bu_⋅(T6)_2_; hydrogen atoms, aryl groups, and solvent molecules are omitted for clarity. The asymmetric unit contains six porphyrins labeled A–F. b) View showing the 27° twist between the mean planes of the two templates. c) Packing diagram with the two enantiomers of *c*-P12_*t*Bu_⋅(T6)_2_ shown in red and blue. d) View of the C–H⋅⋅⋅N contacts between porphyrin units A and F across the external grooves the center of the figure-of-eight. (e and f) Radial projections of porphyrin cores and connecting 1,3-butadiyne linkers in the crystal structures of templated complexes *c*-P12_*t*Bu_⋅(T6)_2_ (e) and *c*-P12_*t*Bu_⋅T6 (f), where *d* is the distance of each atom from the mean plane of the six zinc atoms; *θ* is the angle projected onto this mean plane (see the Supporting Information for detailed description of the construction of these radial plots).

We also analyzed the size and shape of these complexes by using solution-phase small-angle X-ray scattering (SAXS).[19, 20] SAXS data for ***c*****-P12**_***t*****Bu**_**⋅**(**T6**)_2_ and ***c*****-P12**_***t*****Bu**_ in toluene match the simulated pair-distribution functions (PDF) for geometries from molecular mechanics calculations (Figure 3 a,b). The PDF *p*(*r*) represents the probability of finding electron density at separation *r*. In contrast to the template complex, the free nanoring ***c*****-P12**_***t*****Bu**_ is flexible in solution and its SAXS data could only be adequately simulated by using a combination of several elliptical conformations.[9d] The average of the scattering curves from six models is in excellent agreement with the experimental scattering data (Figure 4 b). The Guinier fits[21] calculated from the experimental scattering data for (***l*****-P4**_***t*****Bu**_)_3_**⋅**(**T6**)_2_ are linear in the low-Q region, confirming that the system is monodisperse (Figure 4 c insert). The PDF of (***l*****-P4**_***t*****Bu**_)_3_**⋅**(**T6**)_2_ matches well with the simulated curve, and is similar to that of ***c*****-P12**_***t*****Bu**_**⋅**(**T6**)_2_; the peaks at around 23 and 50 Å correspond to the dimensions from molecular mechanics calculations. The broad PDF function of (***l*****-P4**_***t*****Bu**_)_3_**⋅**(**T6**)_2_ reflects its less regular shape compared with ***c*****-P12**_***t*****Bu**_**⋅**(**T6**)_2_. The radii of gyration *R*_g_ determined for the three structures from the Guinier fit[21] are in good agreement with the values from molecular mechanic calculations (Table 1; MM^+^ force field, HyperChem™).

**Table 1:** Comparison of experimentally determined and calculated radii of gyration *R*_g_

Compound	*R*_g_ (exp, SAXS) [Å]	*R*_g_ (calc) [Å]
***c*****-P12**_***t*****Bu**_**⋅**(**T6**)_2_	20.1	18.2
***c*****-P12**_***t*****Bu**_	23.7	24.8
(***l*****-P4**_***t*****Bu**_)_3_**⋅**(**T6**)_2_	19.0	18.4

### Crystal Structure of *c*-P12_*t*Bu_⋅(T6)_2_[22]

The three-dimensional structure of ***c*****-P12**_***t*****Bu**_**⋅**(**T6**)_2_ was initially deduced from a detailed analysis of the ^1^H NMR and SAXS data.[9d] Crystals of ***c*****-P12**_***t*****Bu**_**⋅**(**T6**)_2_ were grown by slow diffusion of methanol vapor into a solution of ***c*****-P12**_***t*****Bu**_**⋅**(**T6**)_2_ in CHCl_3_ over a period of several days. The best diffraction data were obtained from freshly grown crystals. The crystals contained over 60 % solvent by volume, resulting in weak diffraction. They were assigned to the *C*2/*c* space group with a cell of *a*=117.44(5) Å, *b*=21.009(7) Å, *c*=57.23(2) Å, *α*=90°, *β*=115.385(4)°, *γ*=90°, *V*=127,561 Å^3^. The asymmetric unit contains six porphyrin units (labeled A–F in Figure 4 a), that is, half a molecule of ***c*****-P12**_***t*****Bu**_**⋅**(**T6**)_2_, with a *C*_2_ axis bisecting the molecule at the cross-point of central butadiyne moieties. The distance between the centroids of the two central butadiyne units (along the *C*_2_ axis of the molecule) is 4.24(2) Å and the shortest C⋅⋅⋅C distance between the central carbon atoms is 4.31(2) Å, which is too long for direct van der Waals contact. The torsion angle between these two butadiyne moieties (measured *meso*-centroid-centroid-*meso*) is 74°. This arrangement of the butadiynes is clearly unsuitable for topochemical reaction.[23] There are eight short C–H⋅⋅⋅N contacts across the central groove of the figure-of-eight, between porphyrins A and F, between *tert*-butyl protons and pyrrole nitrogen atoms (Figure 4 d, H⋅⋅⋅N distances: 3.17–3.32(2) Å; C⋅⋅⋅N distances 3.78–4.08(13) Å; C–H⋅⋅⋅N angles: 118–144°). These distances are too long for a classical C–H⋅⋅⋅N hydrogen bond,[24] and they can be classified as C–H⋅⋅⋅π(N) interactions.[25] The distances between the hydrogen atoms to the mean plane of the porphyrin are 2.893–4.00(9) Å. These contacts probably make an insignificant contribution to the energy of the figure-of-eight conformation, but they account for the unusual chemical shift observed for these *tert*-butyl protons (*δ*_H_=−0.64 ppm in CDCl_3_ solution)[9d] and they may explain why the yield for Vernier synthesis of ***c*****-P12_C8_⋅**(**T6**)_2_ is lower than that for the synthesis of ***c*****-P12**_***t*****Bu**_**⋅**(**T6**)_2_. It is easy to see how this type of interaction could become destabilizing when the *t*Bu substituents are changed to larger solubilizing groups.

In the crystal, each molecule of ***c*****-P12**_***t*****Bu**_**⋅**(**T6**)_2_ has *C*_2_ symmetry, with approximate *D*_2_ symmetry. The symmetry in solution is *D*_2_. Both the *C*_2_ and *D*_2_ point groups are chiral, however, the compound crystallizes as a racemate, and each enantiomer constitutes a separate flat layer in which molecules are stacked side-to-side (Figure 4 c). The angle between the mean planes of the two template units is 27° (Figure 4 b).

Comparison of the structures of ***c*****-P6**_***t*****Bu**_**⋅T6**[9c] and ***c*****-P12**_***t*****Bu**_**⋅**(**T6**)_2_ shows that the figure-of-eight topology does not change the size of the six-porphyrin loop. The mean Zn⋅⋅⋅Zn diameter appears to be fixed by the template: 24.35(8) Å in ***c*****-P6**_***t*****Bu**_**⋅T6** vs. 24.36(5) Å in ***c*****-P12**_***t*****Bu**_**⋅**(**T6**)_2_. In contrast, locking two six-porphyrin loops into a figure-of-eight alters the out-of-plane geometry, as shown by the radial projections of the porphyrin cores and 1,3-butadiyne units onto the mean plane of the six zinc centers (Figure 4 e). In the case of ***c*****-P6**_***t*****Bu**_**⋅T6**, the seamless six-porphyrin ring ruffles to adopt a “chair-like” conformation (Figure 4 f),[9c] with alternate butadiynes above and below the plane of the six zinc centers. This chair-conformation only partially persists in the six-porphyrin loop of ***c*****-P12**_***t*****Bu**_**⋅**(**T6**)_2_. Unfortunately, the low resolution of the diffraction data does not allow us to reliably analyze the zinc to pyridine nitrogen bond lengths or bond-length alternation in the 1,3-butadiyne units.

### STM Imaging of *c*-P12_C8_ and *c*-P12_C8_⋅(T6)_2_

**Figure 5:**
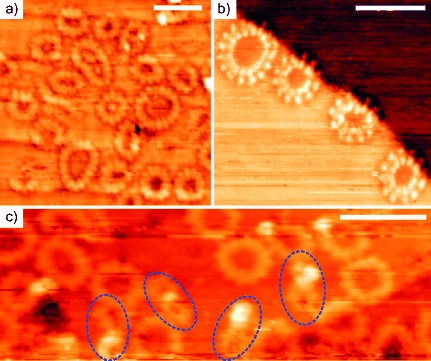
STM imaging of nanorings on a Au(111) surface under UHV. Scale bars: 10 nm. Images a) and b) show samples of *c*-P12_C8_ with some *c*-P16_C8_ impurity. c) *c*-P12_C8_⋅(T6)_2_; most of the molecules of the complex unfold into the free *c*-P12_C8_ during deposition but some intact *c*-P12_C8_⋅(T6)_2_ units are indicated by blue dashed ellipses.

Scanning tunneling microscopy (STM) provides an alternative way to evaluate the structure of ***c*****-P12_C8_** and ***c*****-P12_C8_⋅(T6)_2_** (Figure 5). Molecules were deposited by using an electrospray source, on a Au(111) surface under ultrahigh vacuum, at room temperature, using solutions of the compounds in toluene containing MeOH (5 % by volume).[26] The sample of ***c*****-P12_C8_** used in these experiments was synthesized from ***l*****-P4_C8_** (as described above) without extensive GPC purification and it contained impurities of other cyclic species. The STM images of ***c*****-P12_C8_** showed the presence of many porphyrin nanorings with clearly defined twelve-porphyrin units (Figure 5 a,b). However, the presence of some ***c*****-P16_C8_** was also detected. We attempted to image the ***c*****-P12_C8_⋅**(**T6**)_2_ complex by applying the same imaging conditions used for ***c*****-P12_C8_** (Figure 5 c). Most of the molecules are evident in the form of unfolded ***c*****-P12_C8_**, and the images showed the presence of few intact molecules of ***c*****-P12_C8_⋅(T6)_2_** with clearly defined six-porphyrin loops approximately 2 nm in diameter, consistent with the calculated value of approximately 2 nm. In the case of ***c*****-P12_C8_**, the molecules lie flat on the surface, similar to previously reported STM imaging experiments performed on linear porphyrin oligomers.[26] In contrast, molecules of ***c*****-P12_C8_⋅**(**T6**)_2_ should have the planes of their individual porphyrin units set perpendicular to the gold surface.

### Thermodynamics of Binding of T6 by *c*-P12_*t*Bu_

**Figure 6:**
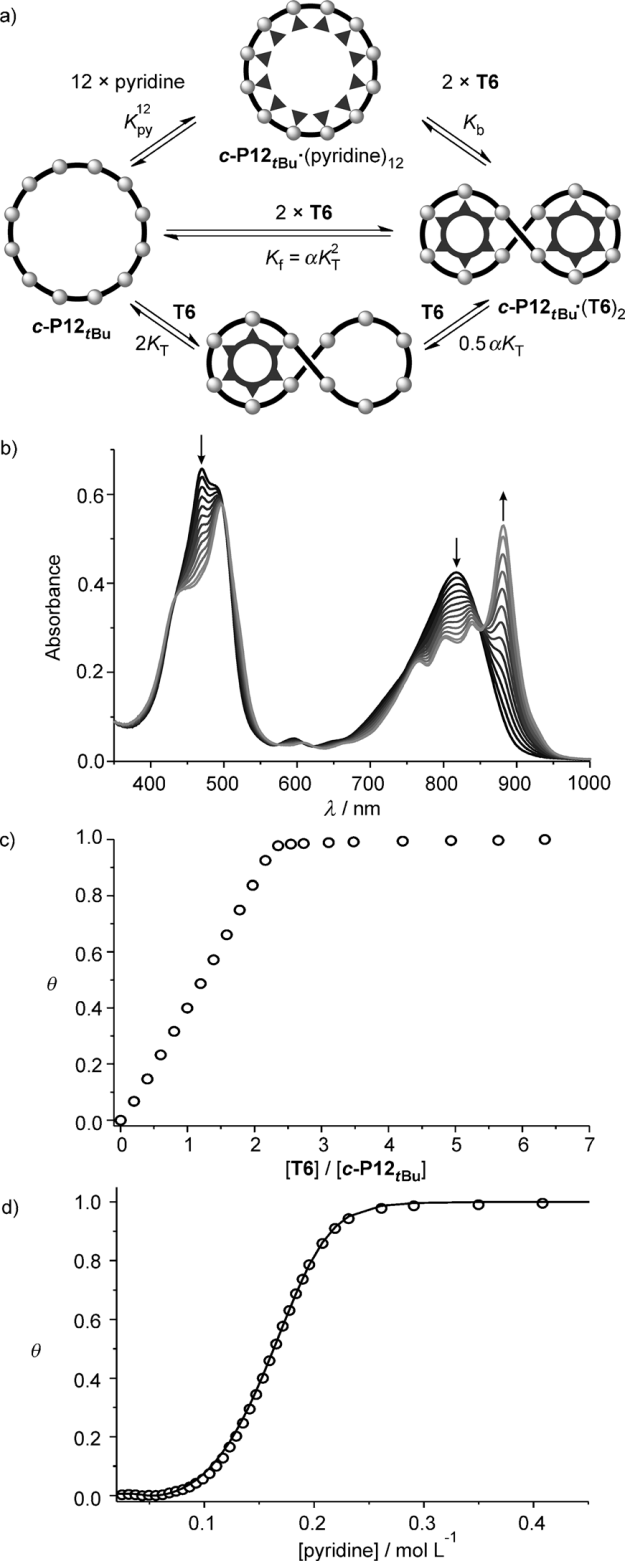
a) Simplified equilibria for the formation of the figure-of-eight complex (*c*-P12_*t*Bu_)⋅(T6)_2_ from *c*-P12_*t*Bu_ and break-up with pyridine. b) Changes in absorption upon addition of T6 to *c*-P12_*t*Bu_ ([c-P12_*t*Bu_]=4.4×10^−7^ m, CHCl_3_, 298 K), and c) fraction of formed *c*-P12_*t*Bu_⋅(T6)_2_ from the difference in absorption Δ*A* at 882–812 nm plotted against the ratio T6/*c*-P12_*t*Bu_. A small amount of pyridine ([pyridine]=6.2×10^−7^ m) was added at the beginning of the titration to disaggregate *c*-P12_*t*Bu_. d) Binding isotherm (black circles) ([*c*-P12_*t*Bu_⋅(T6)_2_]= 5.2×10^−7^ m) derived from absorption data at 883 nm and calculated fit.

**Figure 7:**
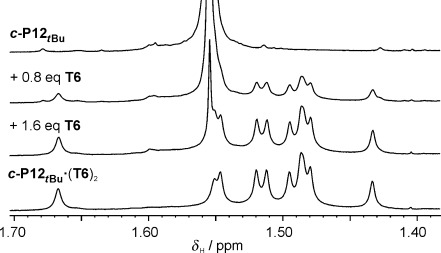
^1^H NMR titration of *c-*P12_*t*Bu_ ([*c-*P12_*t*Bu_]=3.6×10^−4^ m in CDCl_3_, 500 MHz, 298 K) with T6 in CDCl_3_/3 % MeOD. [T6] increases downwards.

When the flexible free nanoring ***c*****-P12**_***t*****Bu**_ binds the **T6** template to form the compact figure-of-eight complex, there is a decrease in the radius of gyration (Table 1) and an increase in the diffusion coefficient (see Figure S3), which are characteristics of a folding event. The cooperativity of this binding process is reminiscent of protein folding. Formation of the 1:2 figure-of-eight ***c*****-P12**_***t*****Bu**_**⋅**(**T6**)_2_ must occur through the formation of a 1:1 complex ***c*****-P12**_***t*****Bu**_**⋅T6** (Figure 6 a). The equilibrium constants of the two events are linked by the interaction parameter *α*, which quantifies the allosteric cooperativity between the binding of the two templates; if *α*=1 then binding of the two **T6** molecules is statistical, if *α*≫1 there is strong positive cooperativity between the two binding events and the intermediate complex ***c*****-P12**_***t*****Bu**_**⋅T6** is not significantly populated. In terms of the allosteric cooperativity between the two template molecules, one would expect the energetic cost of nanoring folding to be mostly paid after the first template is bound. Binding of the second template should be favored because of the preorganization of the binding pocket, giving an interaction parameter *α* greater than 1. This picture of a process with high chelate as well as allosteric cooperativity was confirmed by a ^1^H NMR titration of **T6** into ***c-*****P12**_***t*****Bu**_. A clear transition occurs from the spectrum of ***c*****-P12**_***t*****Bu**_ to that of the figure-of-eight ***c*****-P12**_***t*****Bu**_**⋅**(**T6**)_2_ without any detectable intermediate species. Figure 7 shows the alkyl region of the spectra, which is dominated by the *tert*-butyl singlet at *δ*_H_=1.56 ppm in ***c*****-P12**_***t*****Bu**_. This resonance evolves cleanly into the various *tert*-butyl signals characteristic of the figure-of-eight ***c*****-P12**_***t*****Bu**_**⋅**(**T6**)_2_ complex, without showing any sign of a 1:1 intermediate (although this 1:1 complex is observed by MALDI-TOF MS; Figure 1 d).

The clean all-or-nothing equilibrium between ***c*****-P12**_***t*****Bu**_ and ***c*****-P12**_***t*****Bu**_**⋅**(**T6**)_2_ is also observed at submicromolar concentrations by UV/Vis/NIR titration (Figure 6 b,c). The observation of several isosbestic points indicates the presence of only two absorbing species: the free nanoring and the figure-of-eight complex. The binding isotherm is square and reaches saturation after addition of two equivalents of **T6**, corresponding to the stoichiometry of ***c*****-P12**_***t*****Bu**_**⋅**(**T6**). To quantify the 1:2 cooperativity, it was necessary to first determine the formation constant of the figure-of-eight complex *K*_f_. However, the squareness of the binding isotherm prevents the direct determination of *K*_f_ by means of a formation titration.

Large equilibrium constants can be determined indirectly by competition experiments, as illustrated by the thermodynamic cycle in Figure 6 a.[9a,b, 27] Addition of an excess of the competing ligand pyridine to ***c*****-P12**_***t*****Bu**_**⋅**(**T6**)_2_ will result in displacement of the template molecules and formation of the pyridine complex ***c*****-P12**_***t*****Bu**_**⋅**(pyridine)_12_. The equilibrium constant for this break-up process *K*_b_ and the binding constant of pyridine to ***c*****-P12**_***t*****Bu**_ *K*_py_ can be used to calculate *K*_f_ using Equation (1):


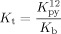
(1)

The binding constant of pyridine with ***c*****-P12**_***t*****Bu**_ is difficult to measure because ***c*****-P12**_***t*****Bu**_ aggregates in the absence of pyridine. The association constant of pyridine with porphyrin monomer ***l*****-P1**_***t*****Bu**_ is expected to be very similar to that with ***c*****-P12**_***t*****Bu**_ and is therefore used as *K*_py_ (*K*_py_=1.0±0.1×10^4^ m^−1^).[9a,b, 27] A large excess of pyridine (ca. 500,000 equivalents) is necessary to completely displace the templates from ***c*****-P12**_***t*****Bu**_**⋅**(**T6**)_2_ at the concentration of a UV/Vis/NIR titration (Figure 6 d). The presence of several isosbestic points (Figure S4) confirms the expected two-state equilibrium, and the sigmoidal binding curve indicates high cooperativity. The equilibrium constant (*K*_b_=7.9±0.8×10^−4^ m^−10^) was determined by fitting the binding isotherm at 883 nm using the program SPECFIT, and the resulting formation constant of the figure-of-eight complex *K*_f_ was 1.8×10^51^ m^−2^. The uncertainty in this number is high because of the error propagation in 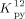 and the value is thus given as log *K*_f_=51.3±0.6.

As shown in Figure 6 a, the formation constant of the figure-of-eight complex *K*_f_ can be expressed by the binding constant of one template *K*_T_ and the interaction parameter *α* accounting for the allosteric cooperativity [Eq. (2)]:


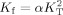
(2)

*K*_T_ depends on the binding constant of one arm of the template *K*_1_ and the average effective molarity *EM* that quantifies the chelate cooperativity [Eq. (3)]:


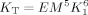
(3)

From Equations (2) and (3), the combined allosteric and chelate cooperativity in the formation of the figure-of-eight complex is given by Equation (4):


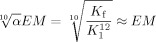
(4)

Since the interaction parameter *α* contributes only in the 10^th^ root to this overall cooperativity, its effect on the value is negligible and the result will be a good approximation of the average effective molarity *EM*.[27] The binding constant of one arm of the template to ***c*****-P12**_***t*****Bu**_ *K*_1_ can be approximated from the binding constant of 4-(phenyl)pyridine to porphyrin monomer ***l*****-P1**_***t*****Bu**_.[9b] With *K*_1_=1.9±0.2×10^4^ m^−1^, the (statistically uncorrected) average effective molarity of figure-of-eight formation is 1.0±0.2 m. It is remarkable that this high effective molarity is comparable to the value of the cyclic octamer-octadentate template complex ***c*****-P8**_***t*****Bu**_**⋅T8** (*EM*=5.4 m) given that ***c*****-P12**_***t*****Bu**_**⋅**(**T6**)_2_ is a three-component assembly and it is significantly more strained. Presumably the first five *EM*s are relatively low because they are associated with most of the strain. The next five *EM*s corresponding to the binding of the second template are probably significantly higher and similar to the values measured for ligand binding in ***c*****-P6**_***t*****Bu**_.[27] The allosteric cooperativity between the two templates originates from the higher effective molarities of the second template.

## Conclusion

The work presented here led to the concept of Vernier template directed synthesis, which appears to be a widely applicable strategy for the preparation of large macrocycles using small, readily available templates.[9e] Our results shed some light on the mechanism of Vernier templating by showing that a Vernier complex (***l*****-P4_C8_**)_3_**⋅**(**T6**)_2_ is formed under the conditions of the coupling reaction.

At first sight, the crystal structure of the figure-of-eight complex ***c*****-P12**_***t*****Bu**_**⋅**(**T6**)_2_ simply confirmed the structure that had already been deduced from NMR and SAXS data. However, on more detailed examination, it revealed several unexpected features, such as the many short C–H⋅⋅⋅N contacts between the *tert*-butyl group of one porphyrin and the central nitrogen atoms of another porphyrin unit. The observation of these interactions reminds us that the side chains are not just solubilizing groups, and that they can influence the conformational behavior of these porphyrin wires. The replacement of these favorable C–H⋅⋅⋅N contacts by unfavorable steric interactions may explain why ***l*****-P4_C8_** undergoes Vernier templated synthesis of ***c*****-P12_C8_** less efficiently than the analogous reaction of ***l*****-P4**_***t*****Bu**_. Coupling of ***l*****-P4_C8_** in the presence of **T6** generates cyclic byproducts such as ***c*****-P8**, ***c*****-P16**, and ***c*****-P24**, which do not appear to be formed from ***l*****-P4**_***t*****Bu**_. The yields of these byproducts are sensitive to the ***l*****-P4_C8_**:**T6** feed ratio, and formation of ***c*****-P12_C8_** is favored by using the ideal 3:2 stoichiometry.

This work illustrates how techniques such as SAXS and STM can play an important role as synthetic supramolecular chemistry moves into the size-domain of protein chemistry. STM is an excellent technique for detecting the presence of larger nanorings, such as ***c-*****P16_C8_** and ***c-*****P24_C8_**, as impurities in ***c-*****P12_C8_**. It was also possible to image the ***c*****-P12_C8_⋅(T6)_2_** figure-of-eight complex, although there was substantial loss of template during electrospray deposition onto the gold surface.

Finally, the results of ^1^H NMR and UV/Vis/NIR titrations show that formation of the ***c*****-P12**_***t*****Bu**_**⋅**(**T6**)_2_ from a ***c*****-P12**_***t*****Bu**_ is a cooperative all-or-nothing folding process, which occurs without detectable amounts of 1:1 intermediates. The formation constant, *K*_f_, of the figure-of-eight complex is 1.8×10^51^ m^−2^ (log *K*_f_=51.3±0.6). It will be interesting to compare the folding processes of larger nanorings such as ***c*****-P16_C8_**, ***c*****-P18_C8_** and ***c*****-P24_C8_**.[9e]

## Supporting Information

As a service to our authors and readers, this journal provides supporting information supplied by the authors. Such materials are peer reviewed and may be re-organized for online delivery, but are not copy-edited or typeset. Technical support issues arising from supporting information (other than missing files) should be addressed to the authors.

miscellaneous_information
