# Supplementary material for: Vernier-Templated Synthesis, Crystal Structure, and Supramolecular Chemistry of a 12-Porphyrin Nanoring
Source: Chemistry. 2014 Aug 25;20(40):12826–34. doi: 10.1002/chem.201403714 (PMC4517159; doi:10.1002/chem.201403714)
Supplement: Supplementary file 1 — miscellaneous_information [file chem0020-12826-sd1.pdf]

CHEMISTRY
A European Journal

Supporting Information

© Copyright Wiley-VCH Verlag GmbH & Co. KGaA, 69451 Weinheim, 2014

Vernier-Templated Synthesis, Crystal Structure, and Supramolecular Chemistry of a 12-Porphyrin Nanoring

Dmitry V. Kondratuk,[a] Johannes K. Sprafke,[a] Melanie C. O'Sullivan,[a] Luis M. A. Perdigao,[b] Alex Saywell,[b] Marc Malfois,[c] James N. O'Shea,[b] Peter H. Beton,[b] Amber L. Thompson,*[a] and Harry L. Anderson*[a]

chem_201403714_sm_miscellaneous_information.pdf

Dmitry V. Kondratuk,[a] Johannes K. Sprafke,[a] Melanie C. O'Sullivan,[a] Luis M. A. Perdigao,[b] Alex Saywell,[b] Marc Malfois,[b] Peter H. Beton,[b] Amber L. Thompson[a] and Harry L. Anderson[a]

[a] Department of Chemistry, University of Oxford, Chemistry Research Laboratory, Oxford OX1 3TA, United Kingdom

[b] School of Physics & Astronomy, University of Nottingham, Nottingham NG7 2RD, United Kingdom

[c] Diamond Light Source Ltd., Harwell Science and Innovation Campus, Didcot OX11 0DE, United Kingdom

Table of Contents

A. Materials and Methods S2
B. Synthetic procedures S3
B1. Known compounds S3
B2. Vernier-templated synthesis of ***c*-P12$_{C8}$** S3
B2a. Coupling of ***l*-P4$_{C8}$** in the presence of **T6** when ***l*-P4$_{C8}$** / **T6** = 1.5 S3
B2b. Coupling of ***l*-P4$_{C8}$** in the presence of **T6** when ***l*-P4$_{C8}$** / **T6** = 1 S4
B3. Vernier-templated synthesis of ***c*-P12$_{t\text{-}Bu}$** S5
B3a. Figure-of-eight complex ***c*-P12$_{t\text{-}Bu}$·(T6)$_2$** S5
B3b. Cyclic porphyrin dodecamer ***c*-P12$_{t\text{-}Bu}$** S6
C. Recycling GPC traces S7
D. DOSY calibration plot S8
E. DOSY spectra of the Vernier complexes S9
F. UV/vis/NIR titration of ***c*-P12$_{t\text{-}Bu}$·(T6)$_2$** S10
G. Solution-phase small angle X-ray scattering (SAXS) S11
H. Crystallography S12
I. References S17

A. Materials and Methods

Dry THF, CH_2Cl_2 and toluene were obtained by passing through alumina under N_2 pressure. All other reagents were used as commercially supplied. Freshly opened $CHCl_3$ (containing 0.5–1.0 % of ethanol as stabilizer, Sigma Aldrich) was used throughout. NMR data were collected at 700 MHz using Bruker AV 700, 500 MHz using a Bruker AVII 500 or at 400 MHz using a Bruker DPX 400 at 298 K. Chemical shifts are quoted as parts per million (ppm) relative to residual $CHCl_3$ at δ 7.27 ppm and coupling constants (J) are reported in Hertz. Room temperature UV-vis-NIR absorbance measurements were recorded with a Perkin-Elmer Lambda 20 photospectrometer. Alumina columns were run using activated basic alumina (Brockmann I, standard grade, ~150 mesh, 58 Å, Sigma Aldrich). Size exclusion chromatography (SEC) was carried out using Bio-Beads S-X1, 200-400 mesh (Bio Rad). The separation of macrocycles was performed using Shimadzu Recycling GPC system, equipped with a LC-20 AD pump, a SPD-20 A UV detector and the line of JAIGEL 3H (20 x 600 mm) and JAIGEL 4H (20 x 600 mm) columns in toluene / 1% pyridine as eluent with a flow rate of 3.5 mL / min.

B. Synthetic Procedures

B1. Known Compounds

Porphyrin tetramer ***l*-P4$_{C8}$**[S1] and hexadentate template **T6**[S2] were synthesized using published procedures.

*B2. Vernier-templated synthesis of **c-P12$_{C8}$***

B2a. Coupling of *l*-P4$_{C8}$ in the presence of T6 when *l*-P4$_{C8}$ / T6 = 1

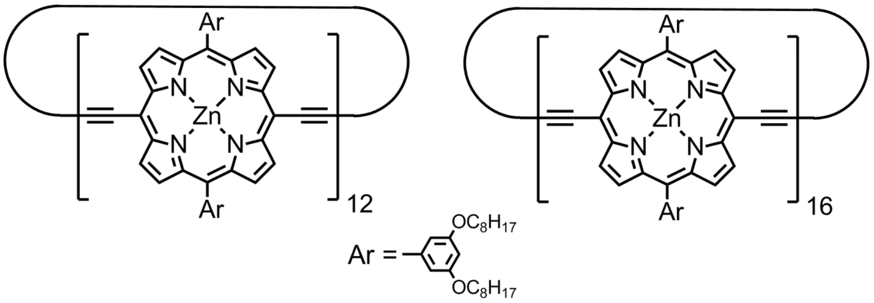

A solution of the deprotected porphyrin tetramer ***l*-P4$_{C8}$** (21.4 mg, 5.0 μmol) in 8.0 mL of CH_2Cl_2 and a solution of the hexadentate template **T6** (5.3 mg, 5.3 μmol) in 2.0 mL of CH_2Cl_2 / 5% MeOH were mixed together and sonicated for 30 min, the solvents removed and the residue dried. The residue was then redissolved in toluene (31.0 mL). A catalyst solution was prepared by dissolving dichlorobis(triphenylphosphine)-palladium(II) (4.6 mg, 6.5 μmol), copper(I) iodide (6.7 mg, 0.033 mmol) and 1,4-benzoquinone (27.2 mg, 0.135 mmol) in the mixture of toluene (3.1 mL) and diisopropylamine (160 μL). The catalyst solution was added to the template and porphyrin tetramer mixture. The reaction mixture was stirred at room temperature for 2 h after which the same amount of the catalysts solution was added and the reaction mixture stirred at 50 °C for 1 h. The reaction mixture was passed through a plug of alumina using $CHCl_3$ as eluent. The crude was further passed over a size exclusion column in $CHCl_3$ / 10% pyridine. Recycling GPC of the porphyrin oligomers mixture afforded 3.5 mg (16 %) of ***c*-P12$_{C8}$** and 1.3 mg (6 %) of ***c*-P16$_{C8}$** as brown solids.

***c*-P12$_{C8}$**: ^{1}H-NMR as reported previously.[S3]

***c*-P16$_{C8}$**: ^{1}H-NMR as reported previously.[S4]

B2b. Coupling of *l*-P4$_{C8}$ in the presence of T6 when *l*-P4$_{C8}$ / T6 = 1.5

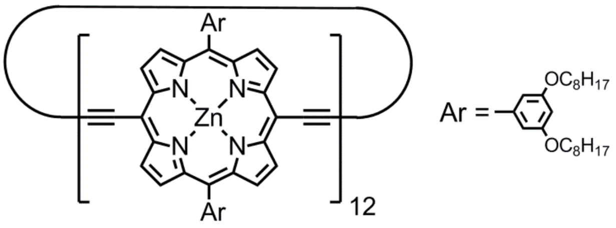

A solution of the deprotected porphyrin tetramer ***l*-P4$_{C8}$** (12.0 mg, 2.8 μmol) in 4.5 mL of CH_2Cl_2 and a solution of the hexadentate template **T6** (1.8 mg, 1.8 μmol) in 1.8 mL of CH_2Cl_2 / 5% MeOH were mixed together and sonicated for 30 min, the solvents removed and the residue dried. The residue was then redissolved in toluene (17.0 mL). A catalyst solution was prepared by dissolving dichlorobis(triphenylphosphine)-palladium(II) (2.6 mg, 3.7 μmol), copper(I) iodide (3.6 mg, 0.019 mmol) and 1,4-benzoquinone (8.3 mg, 0.077 mmol) in the mixture of toluene (1.8 mL) and diisopropylamine (90 μL). The catalyst solution was added to the template and porphyrin tetramer mixture. The reaction mixture was stirred at room temperature for 12 h. The reaction mixture was passed through a plug of alumina using $CHCl_3$ as eluent. The crude was further passed over a size exclusion column in $CHCl_3$ / 10% pyridine. Recycling GPC of the porphyrin oligomers mixture afforded 3.8 mg (32 %) of ***c*-P12$_{C8}$** as a brown solid.

***c*-P12$_{C8}$**: ^{1}H-NMR as reported before.[S3]

*B3. Vernier-templated synthesis of **c-P12$_{t\text{-}Bu}$***

B3a. Figure-of-eight complex *c*-P12$_{t\text{-}Bu}$·(T6)$_2$

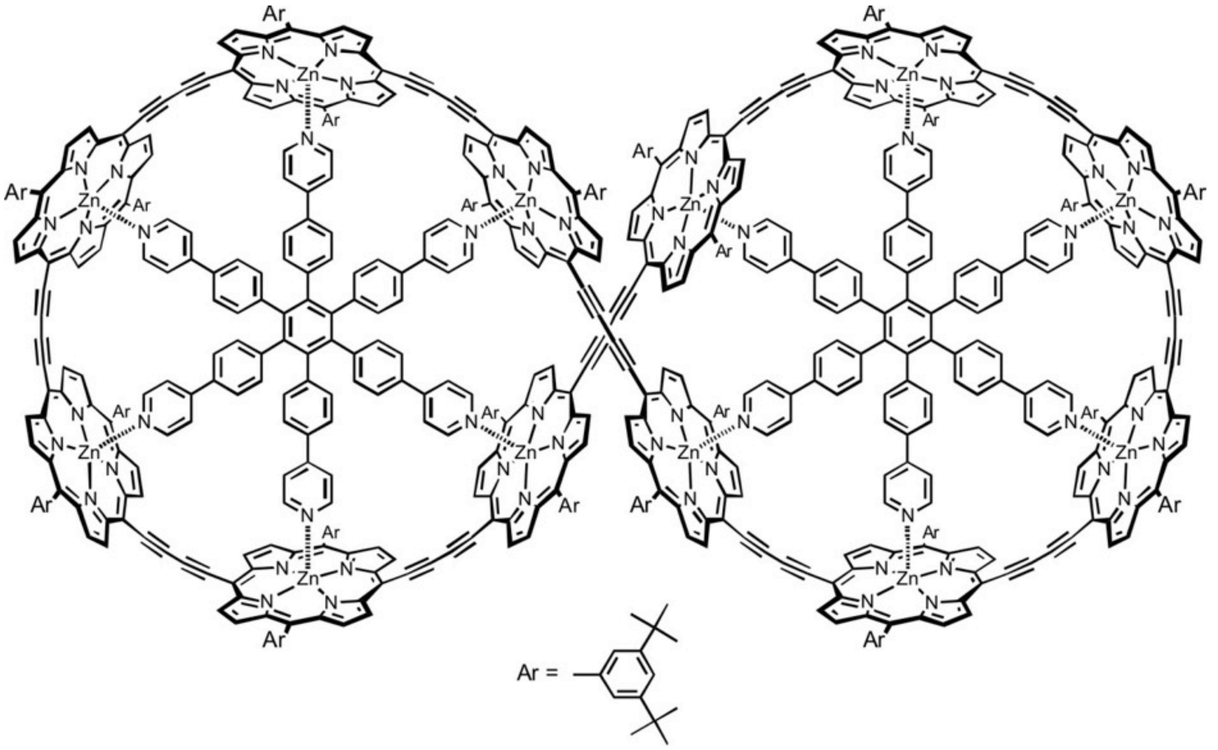

Hexadentate template **T6** (19.5 mg, 19.6 µmol) and deprotected porphyrin tetramer ***l*-P4$_{t\text{-}Bu}$** (61.0 mg, 19.1 µmol) were dissolved in $CHCl_3$ (88 mL) and sonicated for 1 h. A catalyst solution was prepared by dissolving dichlorobis(triphenylphosphine)-palladium(II) (17.7 mg, 25.2 µmol), copper(I) iodide (24.2 mg, 0.127 mmol) and 1,4-benzoquinone (56.1 mg, 0.52 mmol) in $CHCl_3$ (12 mL) and freshly distilled diisopropylamine (610 µL). The catalyst solution was added to the template and porphyrin tetramer mixture. The reaction mixture was stirred at room temperature for 1 h and then for 1.5 h at 50 °C under air. The reaction was passed through a plug of alumina using $CHCl_3$ as eluent. The crude was further purified by size exclusion chromatography on Biobeads SX-1 in toluene. Recrystallization by layer addition (CH_2Cl_2 / MeOH) gave the product as a dark brown solid (29.0 mg, 39%); ^{1}H-NMR (700 MHz, $CDCl_3$): δ_H 10.92 (d, 4H, J = 4.4 Hz, β-H), 10.16 (d, 4H, J = 3.4 Hz, β-H), 9.57–9.53 (m, 32H, β-H), 9.48 (d, 4H, J = 4.3 Hz, β-H), 9.30 (d, 4H, J = 3.6 Hz, β-H), 9.03 (d, 4H, J = 2.9 Hz, β-H), 8.86 (d, 4H, J = 4.4 Hz, β-H), 8.80–8.75 (m, 28H, β-H), 8.72 (d, 4H, J = 3.9 Hz, β-H), 8.32 (d, 4H, J = 3.4 Hz, β-H), 8.11 (s, 4H, Ar-*H*), 8.08 (d, 4H, J = 4.3 Hz, β-H), 8.06–8.02 (m, 16H, Ar-*H*), 7.89 (s, 4H, Ar-*H*), 7.83–7.74 (m, 36H, Ar-*H*), 7.37 (s, 4H, Ar-*H*), 6.98 (s, 4H, Ar-*H*), 6.40 (s, 4H, Ar-*H*), 5.53 (d, 4H, J = 9.7 Hz, -C_6H_4-), 5.49–5.42 (m, 32H, -C_6H_4-), 5.39 (d, 4H, J = 9.2 Hz, -C_6H_4-), 5.27 (d, 4H, J = 9.9 Hz, -C_6H_4-), 5.22 (d, 4H, J = 9.4 Hz, -C_6H_4-), 4.97–4.94 (m, 16H, β-pyridyl), 4.81 (d, 8H, J = 9.4 Hz, β-pyridyl), 2.22–2.19 (m, 24H, α-pyridyl), 1.70 (s, 36H, *t*Bu-CH_3), 1.58 (m, 72H, *t*Bu-CH_3),

1.55–1.54 (m, 72H, *t*Bu-CH_3), 1.53–1.51 (m, 144H, *t*Bu-CH_3), 1.46 (s, 36H, *t*Bu-CH_3), 1.14 (s, 36H, *t*Bu-CH_3), –0.64 (s, 36H, *t*Bu-CH_3); *m/z* (MALDI-ToF) 11554 ($C_{768}H_{696}N_{60}Zn_{12}$, M^+ requires 11551); λ_{max} ($CHCl_3$) / nm (log ε) 497 (5.88), 766 (5.45), 803 (5.54), 882 (5.85), 840 (5.59).

B3b. Cyclic porphyrin dodecamer *c*-P12$_{t\text{-Bu}}$

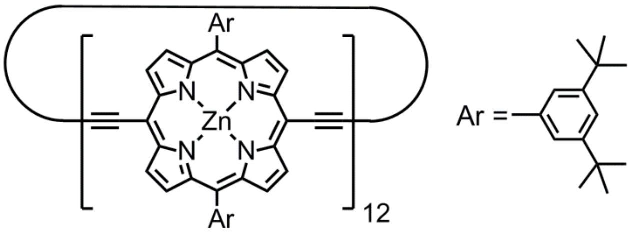

***c*-P12$_{t\text{-Bu}}$·(T6)$_2$** (3.0 mg, 0.26 mmol) was passed over a size exclusion column (Biobeads SX-1) using a mixture of toluene and pyridine (10/1 *v/v*) as eluent. Recrystallization by layer addition ($CHCl_3$ / MeOH) gave the product as a dark brown solid (2.4 mg, 96%); ^{1}H-NMR (500 MHz, $CDCl_3$/1% d_5-pyridine): δ_H 9.84 (d, 48H, J = 4.5 Hz, β-H), 8.95 (d, 48H, J = 4.6 Hz, β-H), 8.05 (d, 48H, J = 1.5 Hz, Ar-*H*), 7.81 (s, 24H, Ar-*H*), 1.56 (s, 432H, *t*Bu-CH_3); ^{13}C-NMR (126 MHz, $CDCl_3$/1% d_5-pyridine): δ_C 153.0, 150.8, 150.0, 148.9, 141.8, 136.3, 133.6, 131.0, 129.4, 128.6, 125.3, 124.0, 121.3, 100.5, 84.3, 35.4, 32.1; *m/z* (MALDI-ToF) 9563 ($C_{624}H_{600}N_{48}Zn_{12}$, M^+ requires 9556); λ_{max} (toluene/1% pyridine) / nm (log ε) 472 (5.86), 488 (shoulder, 5.83), 816 (5.66).

C. Recycling GPC trace

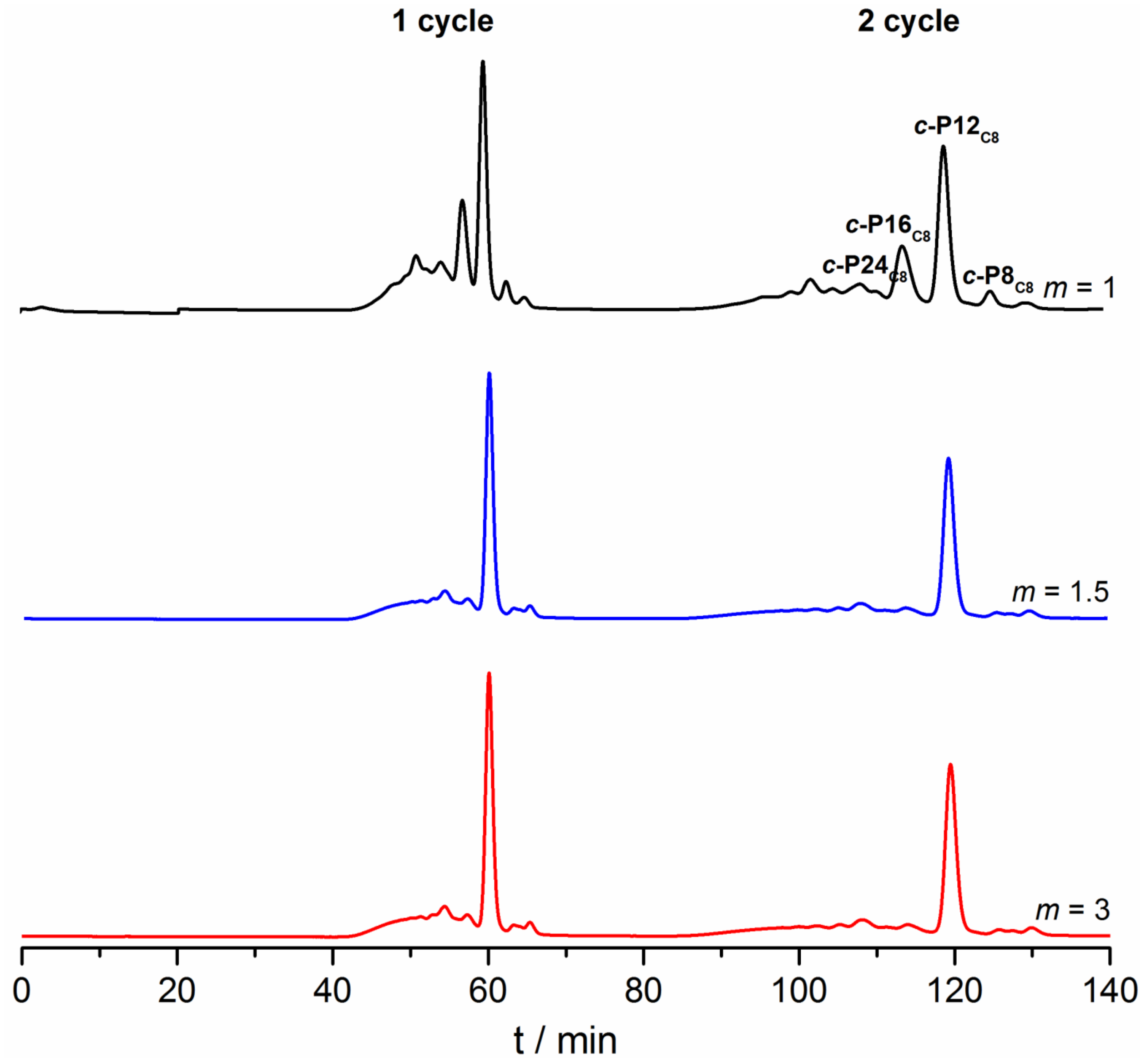

Figure S1. Recycling GPC traces (toluene / 1% pyridine, detection at 500 nm) of the crude reaction mixtures of coupling ***l*-P4**$_{\mathbf{C8}}$ in the presence of **T6** under various ratios ***l*-P4**$_{\mathbf{C8}}$/**T6** = *m*. The mixtures were subjected to two recycling cycles, traces from both cycles are shown. The coupling reagents (the catalysts and 1,4-benzoquinone) and **T6** have been removed by passing through a short alumina column in $CHCl_3$ and the size exclusion column in $CHCl_3$ / 10% pyridine respectively.

D. DOSY calibration plot

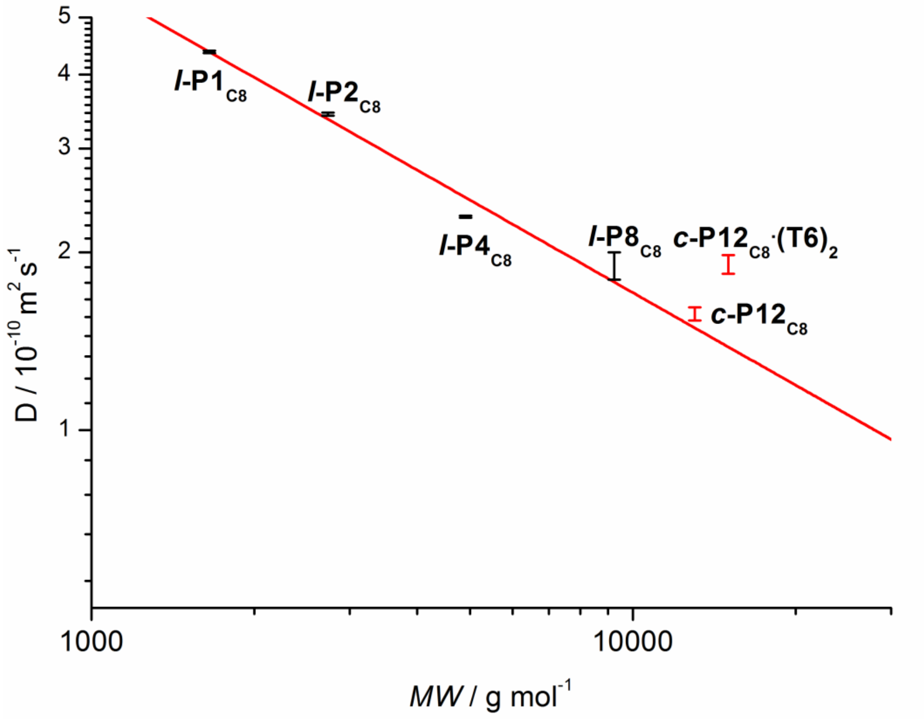

Figure S2. Diffusion coefficients (*D*) of the pyridine complexes of linear oligomers (***l*-P1**$_{C8}$, ***l*-P2**$_{C8}$, ***l*-P4**$_{C8}$, ***l*-P8**$_{C8}$), ***c*-P12**$_{C8}$ and ***c*-P12**$_{C8}$**·(T6)**$_2$ plotted against their molecular weights (*MW*). In all cases the modifications of oligomers bearing octyloxy side chains were used. All DOSY spectra were recorded using the double stimulated echo sequence (DSTE) [S5] with a total diffusion time Δ = 150 ms and gradient pulse duration δ = 4 ms (500 MHz, $CDCl_3$, 298 K).

E. DOSY Spectra of the Vernier complexes

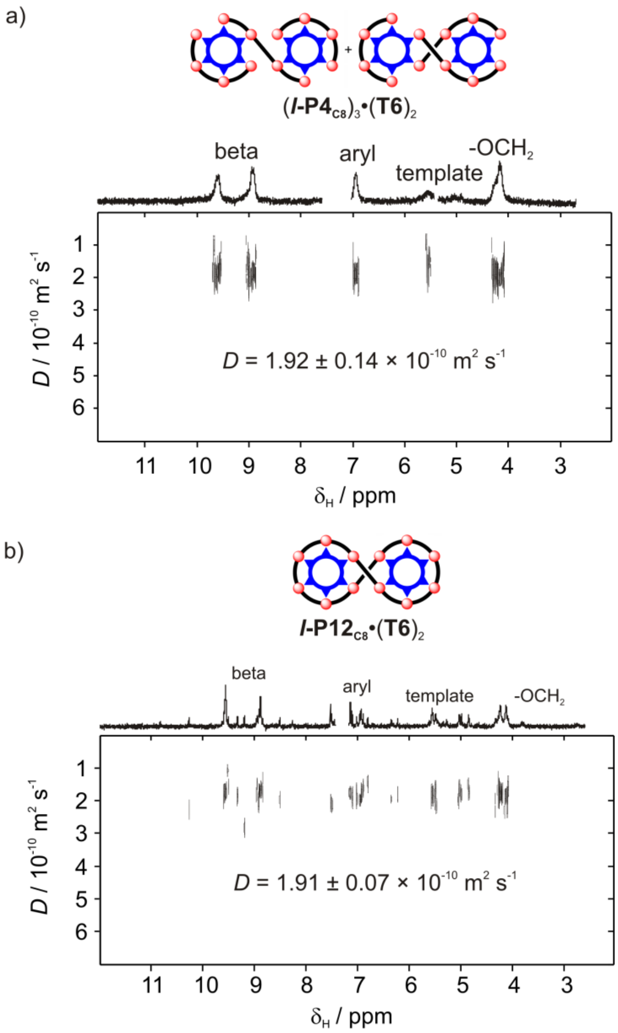

Figure S3. DOSY spectra of (a) the Vernier complex (***l*-P4**$_{\mathbf{C8}}$)$_3$·(**T6**)$_2$ and (b) the figure-of-eight complex (***c*-P12**$_{\mathbf{C8}}$)·(**T6**)$_2$ acquired using the double stimulated echo sequence (DSTE)[S5] with a total diffusion time Δ = 100 ms and gradient pulse duration δ = 4 ms (500 MHz, $CDCl_3$, 298 K). Diffusion coefficients were obtained from fitting porphyrin β and aryl alkoxy signals.

F. UV/vis/NIR titration of c-P12$_{t\text{-Bu}}$·(T6)$_2$

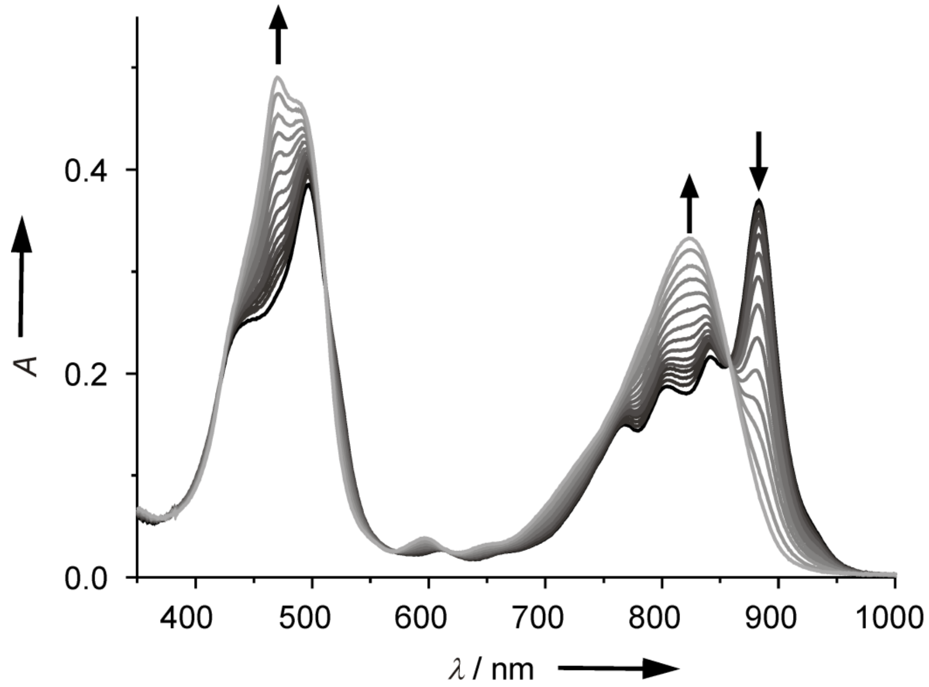

Figure S4. Changes in absorption upon UV/vis/NIR titration of figure-of-eight complex ***c*-P12$_{t\text{-Bu}}$·(T6)$_2$** ([***c*-P12$_{t\text{-Bu}}$·(T6)$_2$**] = 5.2 × 10^{-7} M) with pyridine ($CHCl_3$, 298 K).

G. Solution-phase small-angle X-ray scattering (SAXS)

Synchrotron radiation SAXS data were collected using standard procedures on the I22 beamline at the Diamond Light Source (UK) equipped with a photon counting detector. The beam was focused onto the detector placed at a distance of 1.25 m from the sample cell. The covered range of momentum transfer was $0.03 < q < 1.0$ Å^{-1} ($q = 4\pi \sin(\theta)/\lambda$ where 2θ is the scattering angle and $\lambda = 1.00$ Å is the X-ray wavelength). The data were normalized to the intensity of the incident beam; the scattering of the solvent was subtracted using an in-house program. To check for radiation damage during the SAXS experiment, the data were collected in 10 successive 60 s frames. All SAXS measurements were performed in either toluene or toluene/1% pyridine at known concentrations (~10^{-4} M) in a solution cell with mica windows (1 mm path length). Simulated scattering curves from molecular models were obtained by fitting to the experimental scattering data using the program CRYSOL.[S6] The program GNOM[S7] was used to calculate pair distribution functions and radii of gyration from experimental and simulated scattering data.

H. Crystallography

General crystallographic details for *c*-P12$_{t\text{-Bu}}$•(T6)$_2$.

The sample was very challenging to work with due to the exceptional amount of solvent in the lattice and the fragility of the crystals; the nature of the sample also meant that diffraction was inherently very poor making it challenging to obtain a crystal that diffracted significantly. These properties were intrinsic to the crystals, and despite many recrystallizations and many attempts to collect diffraction data, we struggled to obtain any reliable intensity. In each case, low temperature data were collected at Diamond Light source, beamline I19 (EH1) in order to obtain the best data possible, but the crystals still did not diffract well.

Although crystal growth was not predictable, the best data were obtained on freshly grown crystals; crystals of ***c*-P12$_{t\text{-Bu}}$•(T6)$_2$** were grown by diffusion of methanol into a solution of ***c*-P12$_{t\text{-Bu}}$•(T6)$_2$** in $CHCl_3$ over a period of several days and serendipitously crystals formed the night before the beamtime was available and data were collected. Two crystals from this sample were examined (vide infra) and diffraction data obtained from these were vastly superior to anything else obtained.

The first attempt to collect diffraction data from this optimal sample yielded very poor data, although they were a significant improvement on previous attempts. Earlier experiments had demonstrated that radiation damage was a significant problem. For this reason, the data collection was started with a 360 ° φ-scan, by which time the sample had been damaged beyond further use. Reduction yielded intensities to a maximum of approximately 1.5–2.0 Å resolution. Although an *ab initio* solution was obtained, it was very poor consisting only of the heavy atoms, and as no lighter atoms were found using Fourier methods, it was thought unreliable. However, given the improvement in the quality of the diffraction data, a second crystal was tried.

This second crystal gave better intensities up to approximately 1 Å and *ab initio* structure solution by charge-flipping in the space group P1 using SuperFlip[S8] within CRYSTALS[S9] identified the heavy atom positions. Thus, this resulted in the twelve zinc atoms in the center of the porphyrin motifs as well as some fragments of the light atoms structure (Figure S5). The space group Cc was indicated. Although the unit cell and solutions were approximately consistent, attempts to merge the data reduced its quality, so only the second dataset was used.

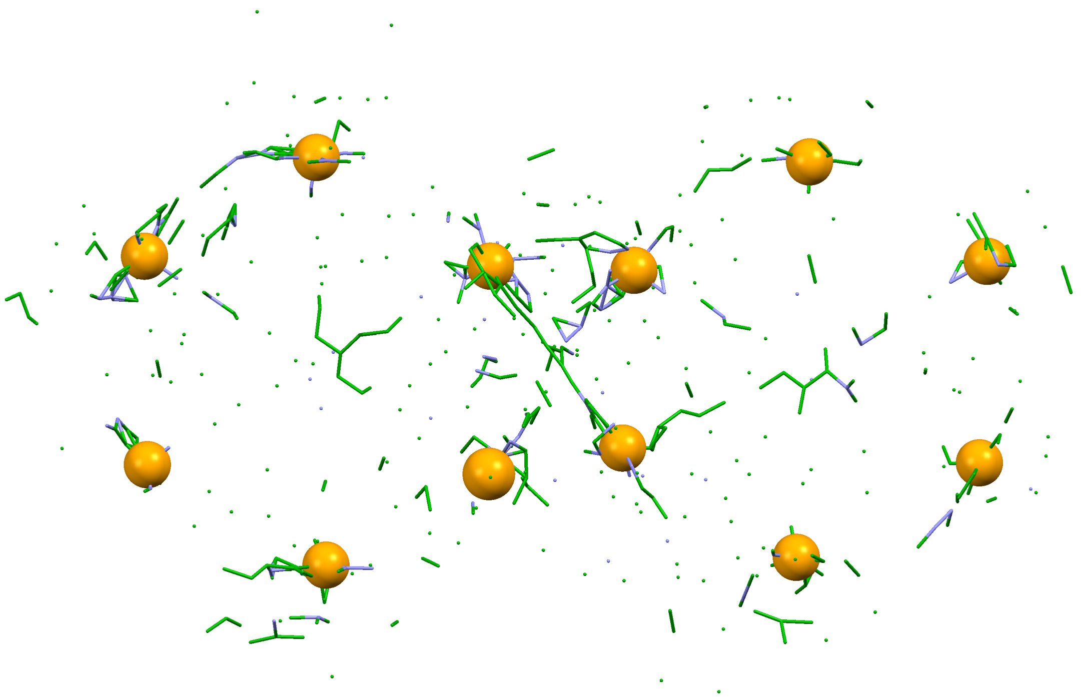

Figure S5. An initial solution for ***c*-P12$_{t\text{-Bu}}$•(T6)**$_2$ showing the heavy atoms in the center of the porphyrin motifs in orange. Although it is clearly a very poor quality structure, it is clear that the "figure of eight" motif is present.

The identification of the heavy atom structure was important from two perspectives. Firstly, the significant electron density was sufficient to provide phasing information. Secondly, the well-known nature of the porphyrin motif and the presence of some fragments of the aromatic systems, meant the positions of the heavy atoms could be used to provide likely positions of some of the missing atoms. The structure was developed using a mixture of difference Fourier methods and structure modeling techniques interspersed with cycles of least-squares refinement with shift-limiting restraints to stabilize the optimization.

Using this method, it was possible to build a model that progressively improved the refinement indicators. During this procedure, a vast number of diffuse peaks were found that did not appear to correspond to the molecular structure. Once the model of ***c*-P12$_{t\text{-Bu}}$•(T6)**$_2$ was complete, further examination of the difference map indicated the presence of diffuse electron density was due to disordered solvent. A preliminary investigation was carried out using PLATON/SQUEEZE[S10] to leave a void from which the electron density was removed and the Fourier transform of the electron density in the void region was added to the model. This led to a significant improvement in the agreement factors.

The atomic skeleton of the model of ***c*-P12$_{t\text{-Bu}}$•(T6)**$_2$ exhibited C_2 symmetry about the center, where the acetylene bridges cross. The presence of this molecular symmetry within the space group *Cc* suggested that symmetry had been omitted. Close examination of the position of the C_2 operator with respect to the remaining structure suggested the correct space group was actually the far more abundant *C2/c*. The model was transformed to give half a molecule in the

asymmetric unit with the central acetyl bridges positioned about the two-fold axis located at ½ *y* ¼. The agreement factors did not significantly deteriorate using this model.

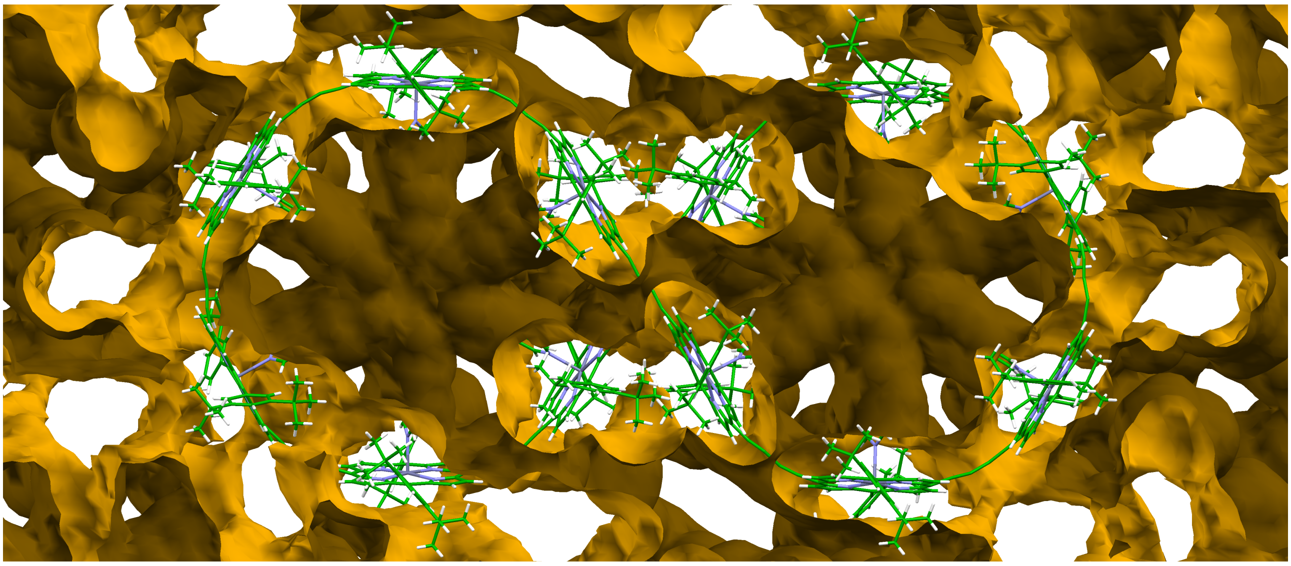

Figure S6. The solvent void surface for one unit cell is shown in orange. The solvent accessible void as calculated by PLATON/SQUEEZE is 62% of the unit cell volume.

Hydrogen atoms were placed at geometric positions and PLATON/SQUEEZE was applied to this model in the new space group. Although this approach is far from ideal since the void region comprised half of the unit cell (Figure S6), the data were not of sufficient quality to treat the problem any other way.

In order to maintain a sensible geometry copious restraints were necessary. In general, rather than restraining bond lengths and angles to absolute values, "SAME" restraints were used extensively making use of the repeated porphyrin motif and its symmetry. Extensive use was also made of thermal similarity and vibrational restraints to allow the displacement parameters to adopt slightly aspherical geometry while ensuring they remained sensible. Best efforts were made to remove the geometric restraints, however, it was necessary to leave a considerable number. The final refinement included a number of "SAME" restraints as well as specific distance and angle restraints keeping the final model in keeping with results from the related structure of the six-porphyrin nanoring.

Although the data are of very low quality giving a challenging refinement requiring extensive restraints with extremely poor final agreement factors, nothing further could be done to improve the data collected.

The asymmetric unit contains 0.5 molecules of ***c*-P12$_{t\text{-Bu}}$·T6** and there is a crystallographic two-fold rotation axis through the center of the molecule. The crystals contained >60% of disordered solvent resulting in weak diffraction. The crystals are assigned to *C2*/*c* space group

with a cell of $a = 117.44(5)$ Å, $b = 21.009(7)$ Å, $c = 57.23(2)$ Å, $\alpha = 90$ °, $\beta = 115.385(4)$ °, $\gamma = 90$ °, $V = 127561$ Å^3. Standard uncertainties are calculated form the full variance covariance or are the sample standard distribution as appropriate.

Radial projections. The radial projections enable a molecule to be visualized 'edge on', as if viewed from the center, and are constructed by plotting displacement (of a given atom, away from some mean plane) against the angle $\theta = \mathrm{R\hat{C}B}$ (where R is an arbitrary reference atom, C is the centroid of the atoms which contribute to the chosen mean plane, and B is a given atom of interest). For the present work, the reference atoms are indicated in Figure S7, with the mean plane of the six zinc centers (in case of figure-of-eight this is six zinc centers of one half), and its centroid, being calculated using Mercury software package. Only atoms of the porphyrin core (no substituent side groups), zinc centers, as well as 1,3-butadiyne linkers were plotted.

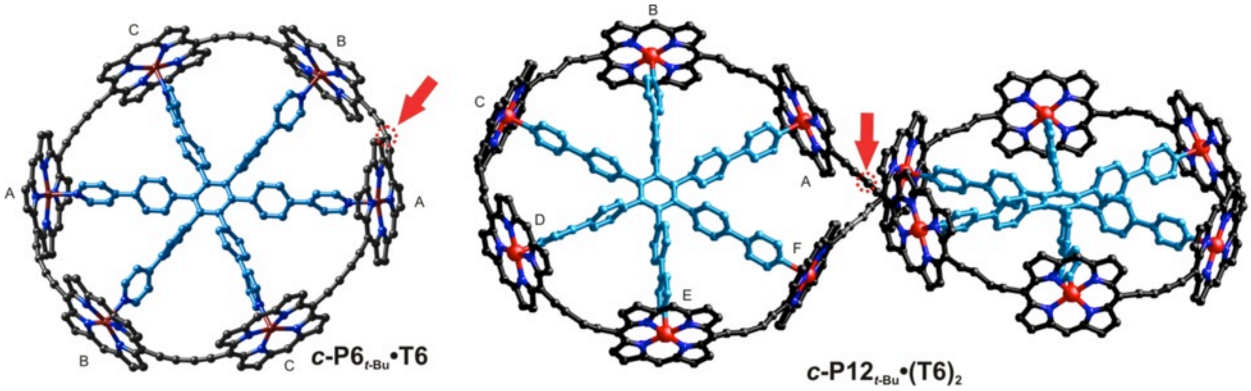

Figure S7. Reference atoms (red arrows) used for building the radial projection plots for crystal structure of ***c*-P6$_{t\text{-Bu}}$•T6**[S11] and ***c*-P12$_{t\text{-Bu}}$ •(T6)$_2$**.

Figure S6 represents the geometrical model used to calculate angles θ.[S11] R and B represent the reference atom and an atom of interest, respectively, whilst C represents the centroid of the 6-zinc atoms mean plane. R' and B' represent the positions of R and B projected onto the mean plane (note that the vectors RR' and BB' are perpendicular to the plane). If BB' were much greater than RR', and R'B' relatively small, then the angle RCB (θ) would be misleadingly large (thereby distorting the data used for graph's abscissa). For this reason, radial projection plots should be constructed using the angle $\mathrm{R\hat{C}B}$ *projected* onto the mean plane (i.e. θ', not θ; Figure S8).

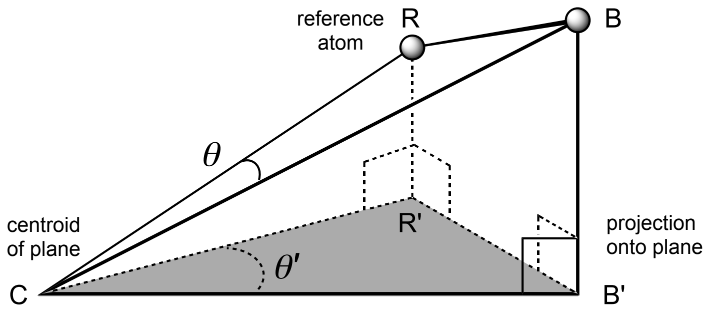

Figure S8. The mathematical model used to construct radial projection plots.

In order to calculate the projected angle R'ĈB' (θ'), the following calculations were performed:

Using Pythagoras' theorem:

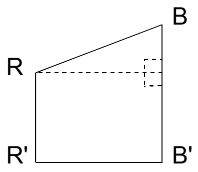

$$RB^2 = R'B'^2 + (BB' - RR')^2$$

$$R'B'^2 = RB^2 - (BB' - RR')^2 \qquad (1)$$

Using the cosine rule:

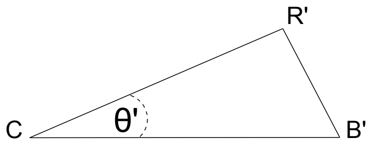

$$R'B'^2 = CR'^2 + CB'^2 - 2(CR'\,CB')(\cos\theta')$$

$$\cos\theta' = \frac{CR'^2 + CB'^2 - R'B'^2}{2(CR'\,CB')} \qquad (2)$$

Substituting (1) into (2):

$$\cos\theta' = \frac{CR'^2 + CB'^2 - [RB^2 - (BB' - RR')^2]}{2(CR'\,CB')}$$

$$\cos\theta' = \frac{CR'^2 + CB'^2 - RB^2 + (BB' - RR')^2}{2(CR'\,CB')} \qquad (3)$$

Using Pythagoras' theorem to obtain CR' and CB' in terms of measurable parameters:

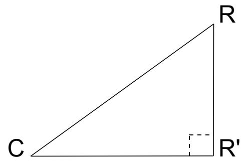

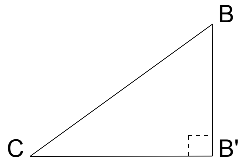

$$CR^2 = RR'^2 + CR'^2$$

$$CR' = (CR^2 - RR'^2)^{1/2} \qquad (4)$$

$$CB^2 = BB'^2 + CB'^2$$

$$CB' = (CB^2 - BB'^2)^{1/2} \qquad (5)$$

Substituting (4) and (5) into (3):

$$\cos\theta' = \frac{[(CR^2 - RR'^2)^{1/2}]^2 + [(CB^2 - BB'^2)^{1/2}]^2 - RB^2 + (BB' - RR')^2}{2\,(CR^2 - RR'^2)^{1/2}(CB^2 - BB'^2)^{1/2}}$$

$$\cos\theta' = \frac{CR^2 - RR'^2 + CB^2 - BB'^2 - RB^2 + (BB' - RR')^2}{2\,(CR^2 - RR'^2)^{1/2}(CB^2 - BB'^2)^{1/2}}$$

$$\cos\theta' = \frac{CR^2 - RR'^2 + CB^2 - BB'^2 - RB^2 + BB'^2 - BB'\,RR' - RR'\,BB' + RR'^2}{2\,(CR^2 - RR'^2)^{1/2}(CB^2 - BB'^2)^{1/2}}$$

$$\cos\theta' = \frac{CR^2 + CB^2 - RB^2 - 2BB'\,RR'}{2\,(CR^2 - RR'^2)^{1/2}(CB^2 - BB'^2)^{1/2}} \qquad (6)$$

All of the parameters on the right hand side of equation (6) are known, thereby allowing the calculation of θ'.

I. References

S1 P. N. Taylor, H. L. Anderson, *J. Am. Chem. Soc.* **1999**, *121*, 11538–11545.

S2 M. Hoffmann, J. Kärnbratt, M.-H. Chang, L. M. Herz, B. Albinsson, H. L. Anderson, *Angew. Chem. Int. Ed.* **2008**, *47*, 4993–4996.

S3 M. C. O’Sullivan, J. K. Sprafke, D. V. Kondratuk, C. Rinfray, T. D. W. Claridge, A. Saywell, M. O. Blunt, J. N. O’Shea, P. H. Beton, M. Malfois, H. L. Anderson, *Nature* **2011**, *469*, 72–75.

S4 D.V. Kondratuk, L. M. A. Perdigao, M. C. O’Sullivan, S. Svatek, G. Smith, J. N. O’Shea, P. H. Beton, H. L. Anderson, *Angew. Chem. Int. Ed.* **2012**, *51*, 6696–6699.

S5 A. Jerschow, N. Muller, *J. Magn. Reson.* **1997**, *125*, 372–375.

S6 D. I. Svergun, C. Berbato, M. H. J. Koch, *J. Appl. Cryst.* **1995**, *28*, 768–773.

S7 D. I. Svergun, *J. Appl. Cryst.* **1992**, *25*, 495–503.

S8 L. Platinus, G. J. Chapuis, *Appl. Cryst.* **2007**, *40*, 786–790.

S9 P. W. Betteridge, J. R. Carruthers, R. I. Cooper, K. Prout, D. J. Watkin, *J. Appl. Cryst.* **2003**, *36*, 1487.

S10 A. L. Spek, *J. Appl. Cryst.* **2003**, *36*, 7–13; P. van der Sluis, A. L. Spek, *Acta Cryst.* **1990**, *A46*, 194–201.

S11 M. J. Smith, D.Phil. thesis, Oxford, 2004.
